# The First Successful Transcatheter Closure of an Inferior Sinus Venosus Defect with Anomalous Drainage of the Right Lower Pulmonary Vein Using Bare and Covered Stents: A Single-Case Report

**DOI:** 10.1155/2022/9392811

**Published:** 2022-10-13

**Authors:** Kritvikrom Durongpisitkul, Paweena Chungsomprasong, Porntip Panjasamanvong, Somrach Thamtorawat

**Affiliations:** ^1^Division of Pediatric Cardiology, Department of Pediatrics, Faculty of Medicine Siriraj Hospital, Mahidol University, Bangkok, Thailand; ^2^Siriraj Piyamaharajkarun Hospital, Mahidol University, Bangkok, Thailand; ^3^Division of Diagnostic Radiology, Department of Radiology, Faculty of Medicine Siriraj Hospital, Mahidol University, Bangkok, Thailand

## Abstract

Inferior sinus venosus defect (SVD) is less common than a superior one. The lower edge of the defect straddles the orifice of the inferior vena cava, and this makes surgical repair via bicaval cannulation a technical challenge. The orifice of the unroofed right pulmonary vein is caused by the interatrial communication in sinus venosus defects which results in partial anomalous pulmonary vein drainage (PAPVD). Novel transcatheter closure of a superior SVD has recently been described; however, transcatheter closure of an inferior SVD has not yet been reported in the published literature. Here, we report the first successful transcatheter closure of an inferior SVD with bare and covered stents and the rerouting of a PAPVD into the left atrium to avoid occlusion of the hepatic veins. In this single-case report, we carefully describe the planning process, how the procedure was performed, and the steps taken to recapture and reposition a migrated stent. Careful patient selection and intensive assessment of pulmonary and hepatic vein anatomy before and during the procedure were necessary to achieve a successful outcome.

## 1. Introduction

Ectopic or incomplete resorption of the sinus venosus results in deficiency of the wall that separates the right pulmonary veins from the superior vena cava (SVC), inferior vena cava (IVC), and right atrium resulting in sinus venosus defect (SVD). A defect of this wall leads to abnormal pulmonary venous drainage to the RA which is partial anomalous pulmonary vein drainage (PAPVD) with either the superior and/or middle-right pulmonary vein and less commonly associated with the right lower pulmonary vein (RLPV). Sinus venosus defects are more commonly found at the SVC-RA junction (SVC type) than at the IVC-RA junction (IVC type). Sometimes the defect extends to the right atrium (RA type). Novel techniques of transcatheter closure of superior SVD were reported. The first 5 cases of transcatheter closure of superior SVD were done by Abdullah et al. in 2011 [1] and presented in the conference of CSI-Frankfurt 2013. Later, Garg et al. published the one case report transcatheter closure of an SVD using a stent [2].

Since then, different stents and implantation techniques have been reported [1, 3, 4]. An inferior SVD is less common and is characterized by the lower edge of the defect straddling the orifice of the IVC, and this makes repair via bicaval cannulation a technical challenge. A similar principle was used to describe the transcatheter closure of ASD with absent inferior rim using stent with or without device [5]. To the best of our knowledge, there is only one report of successful transcatheter closure of an inferior SVD, and that closure was achieved using a device [6]. Here, we report the first successful transcatheter closure of an inferior SVD with anomalous drainage of the right lower pulmonary vein using bare and covered stents.

## 2. Case Report

**Figure 1:**
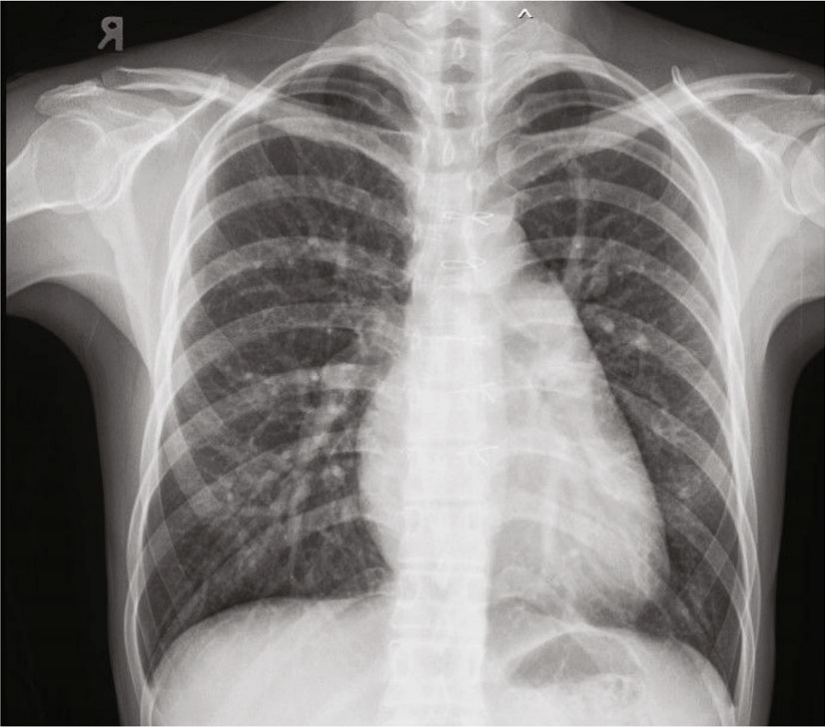
Plain radiograph of the chest showing mild cardiomegaly with left atrialization and slight increase in pulmonary blood flow.

**Figure 2:**
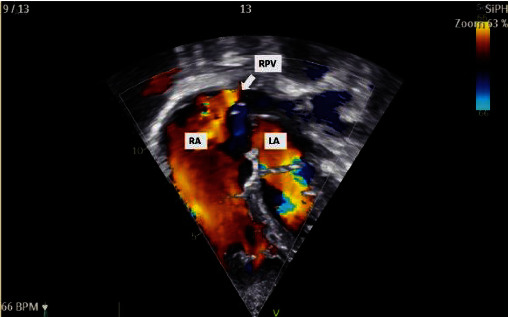
Echocardiography showing a four-chamber view with partial anomalous right pulmonary venous drainage with blood flow from the right lower pulmonary vein to inferior vena cava. LA: left atrium; RA: right atrium; RPV: right pulmonary vein.

**Figure 3:**
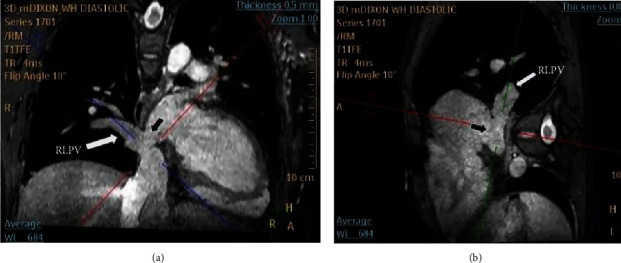
Cardiac magnetic resonance imaging shows a residual sinus venosus defect (black arrow) between the right lower pulmonary vein (RLPV) and inferior vena cava. (a) Coronal view and (b) sagittal view from the left side. Anomalous right lower pulmonary vein drainage can also be observed.

A 29-year-old Thai female had dyspnea during exertion with past history of surgical atrial septal defect (ASD) closure at another hospital at 8 years old. On cardiac examination, a 2/6 systolic ejection murmur was detected. CXR taken at our hospital showed mild cardiomegaly (Figure 1). Two-dimensional transthoracic echocardiography revealed marked right atrial and right ventricular enlargement with left to right shunt via an inferior SVD with a suspected PAPVD (Figure 2). Cardiac magnetic resonance (CMR) imaging confirmed the diagnosis of an inferior SVD that measured 17.2 mm in length with partial anomalous pulmonary venous drainage (PAPVD) of the RLPV into the IVC (Figures 3(a) and 3(b)). We suspected that diagnosis of secundum ASD previously did not include an inferior PAPVD in this patient; hence, the surgical procedure did not incorporate PAPVD. The patient's right ventricle (RV) was severely dilated with a right ventricular end-diastolic volume index (RVEDVi) of 172 ml/m^2^, RV ejection fraction (RVEF) of 59%, a left ventricular end-diastolic volume index (LVEDVi) of 66.2 ml/m^2^, and an LVEF of 63%. The QP : QS measurement was 2.17 : 1. It was clear that the shunt was significant and that closure was required. Due to our patient's previous history of open-heart surgery, we decided to opt for transcatheter closure instead of reoperation.

**Figure 4:**
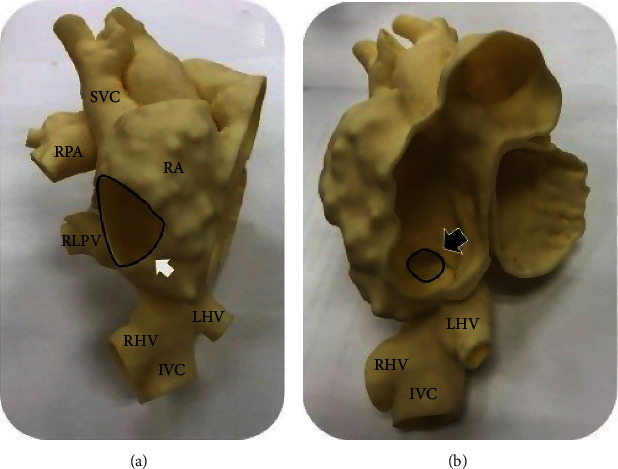
Anterior aspect of a 3-dimensional model from cardiac magnetic resonance imaging. (a) The superior vena cava- (SVC-) right atrium (RA) junction, right pulmonary artery (RPA), right lower pulmonary vein (RLPV), right hepatic vein (RHV), left hepatic vein (LHV), and inferior vena cava (IVC) can be observed. An opening was artificially created (white arrow) to facilitate a simulated view into the lateral aspect of the RA. (b) A sinus venosus defect was identified at the IVC-RA junction (black arrow).

**Figure 5:**
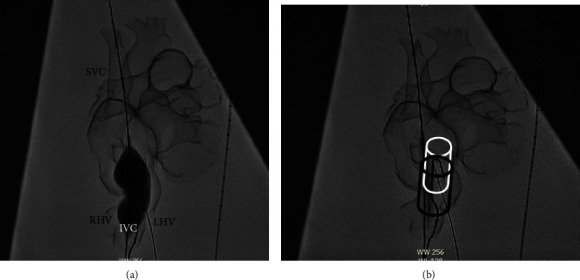
A 3-dimensional model was created to simulate balloon sizing in the inferior vena cava (IVC) (a). The critical point at the IVC-right atrium junction was 20.3 mm. A decision was made to position a 24 mm anchored bare stent (black outlined stent) at this location (b). A long wire was placed in the superior vena cava (SVC), and a short wire was placed in both the left hepatic vein (LHV) and the IVC. The aim of the placement of the bare stent (black outlined stent) and the subsequently positioned covered stent (white outlined stent) was to cover the sinus venosus defect and to ensure no obstruction of blood flow in the LHV and the right hepatic vein (RHV).

Similar to a superior SVD, a PAPVD in an inferior SVD is caused by a defect in the posterior wall of the IVC and in the anterior wall of the RLPV. We hypothesized that careful placement of one or more covered stents in the IVC could both close the defect and facilitate normal RLPV drainage into the LA. The challenge was where to position the covered stent in the IVC without compromising hepatic venous drainage. Based on three-dimensional (3D) reconstruction of the respiratory-navigated and electrocardiography- (ECG-) gated Dixon CMR sequence, a 3D-printed model (Figure 4(a)) was used to simulate the landing zone for each bare and covered stent deployment, including the preferable angle of angiography. The model was used to evaluate an anterior view of the RA (Figure 4(a)) and by looking into an opening that was artificially cut into the lateral wall of the RA to directly visualize the SVD (Figure 4(b)). An interventional radiologist was invited to our routine heart team meeting. To prevent hepatic vein obstruction, it was decided to anchor bare stent in the IVC just above the left hepatic vein orifice. The 3D model was also used for simulation of balloon sizing to measure the anchoring area diameter of 20.27 mm (Figure 5(a)). The aim of the covered stent was to cover the superior border of the ASD (Figure 5(b)). The length of the stent was decided according to the distance between the features of the IVC just above the entrance of the left hepatic vein and the upper rim of the SVD defect, which was measured to be 60 mm. Since the longest BeGraft ePTFE covered stent (BGA4824_2; Bentley InnoMed GmbH, Hechingen, Germany) is 48 mm in length, we decided to use two overlapping stents, including the aforementioned BeGraft covered stent and one CP8Z45 bare stent (NuMED for Children, Orlando, FL, USA) that is 45 mm in length and that was used as the primary anchoring stent outside of the BeGraft stent.

**Figure 6:**
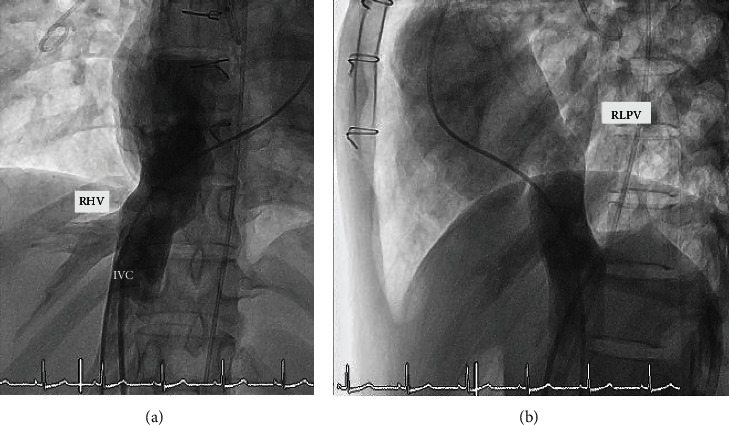
Angiography was performed in the inferior vena cava (IVC). The (a) anterior and (b) lateral views show the connection between the right lower pulmonary vein (RLPV) and the right atrium. The decision was made to place the lower end of the covered stent above the entrance of the left and right hepatic veins (RHV).

The procedure was performed under general anesthesia in the cardiac catheterization lab. Sheathes were inserted via the right femoral vein and the right femoral artery. Fifty units/kg of unfractionated heparin and ceftriaxone were given intravenously. The activated clotting time (ACT) was monitored and maintained within 200-250 seconds. Angiography was performed in the IVC to visualize hepatic vein entrance into the IVC (Figures 6(a) and 6(b)).

**Figure 7:**
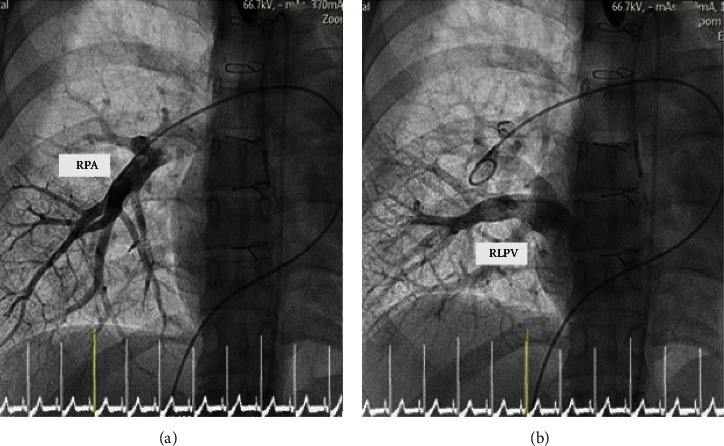
The (a) arterial phase and (b) levo phase of right pulmonary artery (RPA) angiography shows drainage of the right lower pulmonary vein (RLPV) into the right atrium.

**Figure 8:**
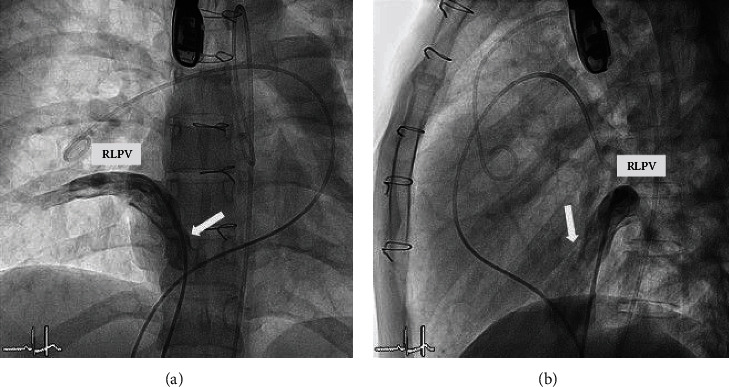
Right lower pulmonary vein (RLPV) angiography was performed simultaneous both (a) anterior view and (b) lateral view of the right atrium. The drainage of the RLPV into the right atrium (white arrow) was also shown in both views. (a) The decision was made to place the upper part of the covered stent and anterior and above the entrance of this RLPV (white arrow) to direct the pulmonary venous blood return to the left atrium.

Right pulmonary artery angiography was performed to visualize both the arterial phase (Figure 7(a)) and the levo phase (Figure 7(b)) of RLPV drainage. RLPV venography was performed in the anteroposterior (AP) (Figure 8(a)) and lateral (Figure 8(b)) views to determine the location of the RLPV relative to the posterior wall of the RA. The site of the entrance of the RLPV into the IVC and the lower end of the SVD were also profiled in this angiogram (Figures 7 and 8).

**Figure 9:**
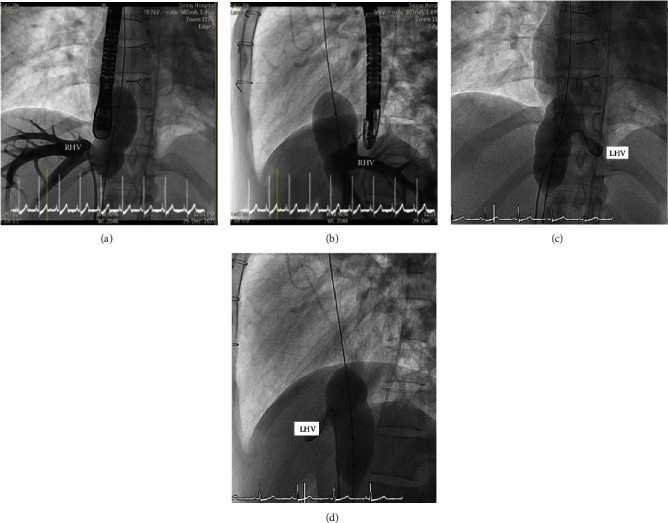
Balloon sizing of the sinus venosus defect that was performed at the inferior vena cava (IVC)-right atrium junction ((a) anterior and (b) lateral views on angiography) showed the right hepatic vein (RHV) to be occluded by the balloon. During the same occlusion, we placed a catheter in the left hepatic vein (LHV) and simultaneously injected contrast showing the LHV connection ((c) anterior and (d) lateral views).

**Figure 10:**
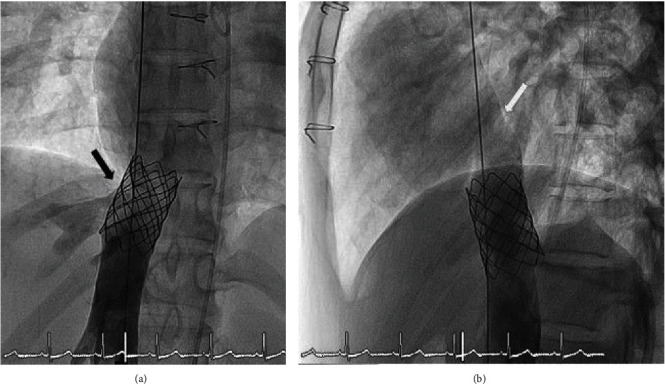
The bare stent, which was placed first, was positioned at the inferior vena cava-right atrium junction ((a) anteroposterior and (b) lateral views on angiography). The decision was then made to position the lower end of the planned covered stent, which will be placed later, above the entrances of the right and also left hepatic veins (black arrow), and that the covered stent would overlap with the bare stent approximately 50%. It was also noted that the distal part of this covered stents should position just above the entrance of the right lower pulmonary vein (white arrow).

**Figure 11:**
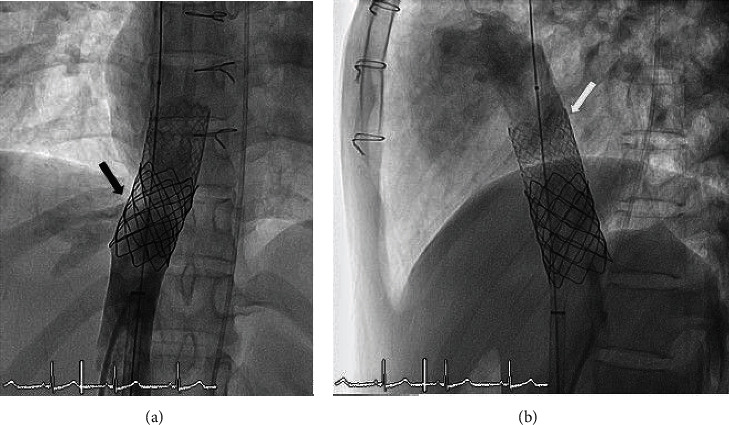
The covered stent (white arrow), which was placed second, is positioned superior to the anchored bare stent (black arrow) in the inferior vena cava ((a) anteroposterior and (b) lateral views on angiography). The placement of covered stent was supposed to be above the entrance of the right lower pulmonary vein (RLPV) but at the expense of overlapping segment because the bare stent was placed lower enough into the IVC. This can be compared with final position of the covered stent after repositioning almost covering the entrance of RLPV but lower than the first position (Figure 15). The stitch can be used as a guide line.

We used the largest available size balloon which is a 30 mm PTS-X balloon (NuMED for Children, Orlando, FL, USA) to generate a 1 atm pressure for sizing. A simultaneous contrast injection was performed at both right (Figures 9(a) and 9(b)) and left (Figures 9(c) and 9(d)) hepatic veins. A higher-pressure balloon will be needed during implantation of the stent. The anchoring CP8Z45 bare stent over a 22 mm × 50 mm Balloon-in-Balloon (BIB) balloon catheter (NuMED for Children, Orlando, FL, USA) was successfully deployed and redilated using a larger 24 mm × 40 mm Z-Med II balloon (NuMED for Children, Orlando, FL, USA) that can expand up to 24 mm in diameter inside the IVC. After stent placement, right hepatic vein flow could freely drain into the stent (Figures 10(a) and 10(b)). The decision was made to place the lower end of the covered stent above the entrance of the hepatic veins, but the bare stent had been positioned lower down than proper position making the overlapping segment shorter than 50% as shown in Figure 10 as angiogram is more practical and informative during the procedure than CMR, cardiac CT, or 3D printing. Placement of the planned subsequent covered stent using a 24 mm × 48 mm BeGraft ePTFE Covered Stent (AccuPath Medical Technologies Co., Ltd., Shanghai, China) was positioned at the superior aspect of the primary anchoring stent at just above the left hepatic vein drainage. We estimated that this positioning would allow enough distance above the openings of both the right and left hepatic veins (Figure 10). However, this reduced the overlap between stents, which compromised the stability of the covered stent. Nonetheless, the covered stent was finally deployed at the intended position (Figures 11(a) and 11(b)).

**Figure 12:**
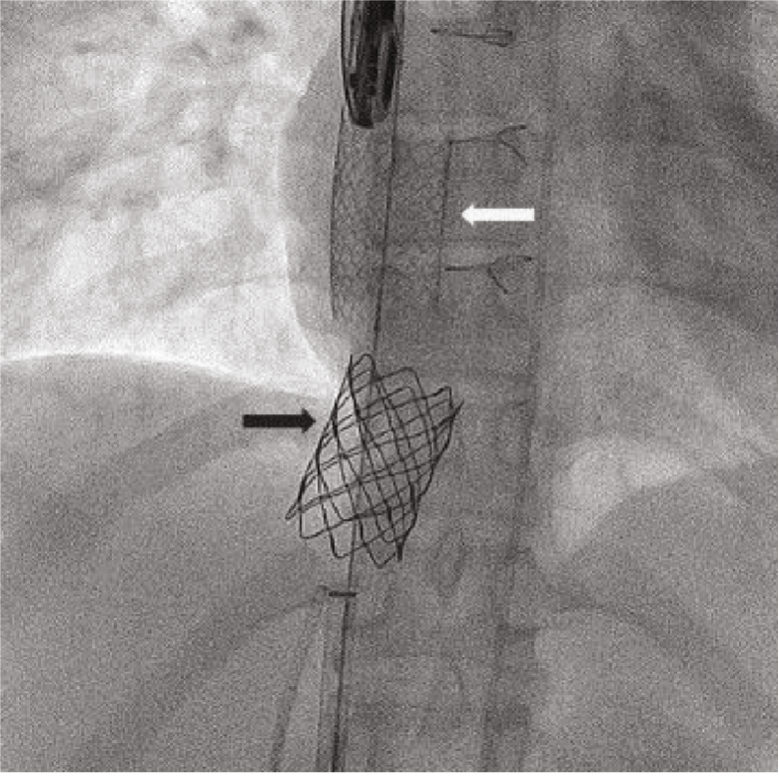
The covered stent unexpectedly embolized into the right atrium as visualized on anterior view angiography of the right atrium. The black arrow and white arrow indicate the position of the bare stent and the covered stent, respectively.

**Figure 13:**
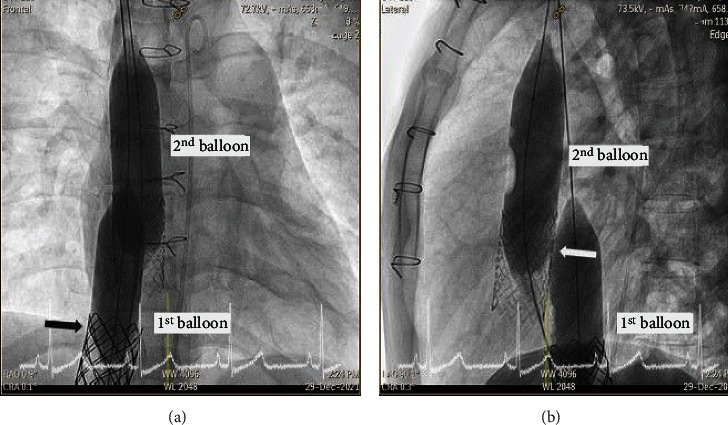
The balloon inside the covered stent was inflated in an attempt to pull down and reposition the embolized covered stent, but that attempt was unsuccessful. We then deployed another 24 mm × 40 mm Z-Med balloon in an attempt to use an external force to squeeze or pushing the embolized covered stent to a smaller diameter to be able to pull back inside first stent, but that was also unsuccessful due to the strong radial force of the covered graft (a, b). The black arrow and white arrow indicate the position of the bare stent and the covered stent, respectively.

The covered stent then unexpectedly migrated into the RA shortly after it was deployed (Figure 12). The first attempt to reposition the covered stent using a balloon inflated inside the stent to try to pull it backwards and back into position was unsuccessful. We then deployed a second 24 mm × 40 mm Z-Med II balloon in an attempt to use an external force to squeeze or push the migrated covered stent to a smaller diameter to be able to pull back inside first stent, squeeze; however, this attempt was also unsuccessful due to the strong radial force of the covered stent (Figures 13(a) and 13(b)).

**Figure 14:**
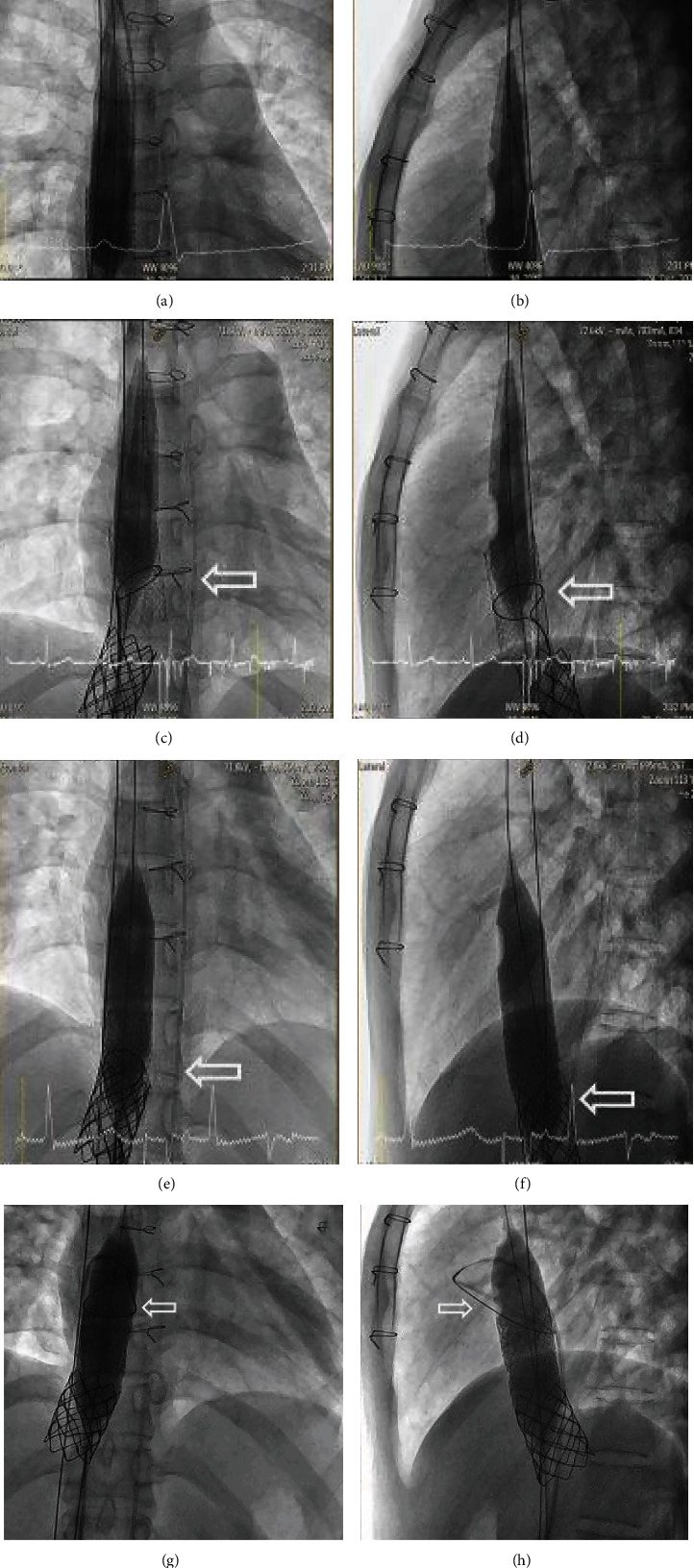
A 35 mm Amplatz Gooseneck snare was deployed from a distal Amplatz Super Stiff wire (a, b). The snare was used to grasp the middle of the embolized covered stent and to pull it back inside the anchored bare stent (c, d); however, we were not able to release the snare once the covered stent was back inside the bare stent (e, f). We then redeployed the snare at a more distal position and then pulled the embolized stent with balloon inflated in the more distal position to coaxial both stents and eventually released snare (g, h).

**Figure 15:**
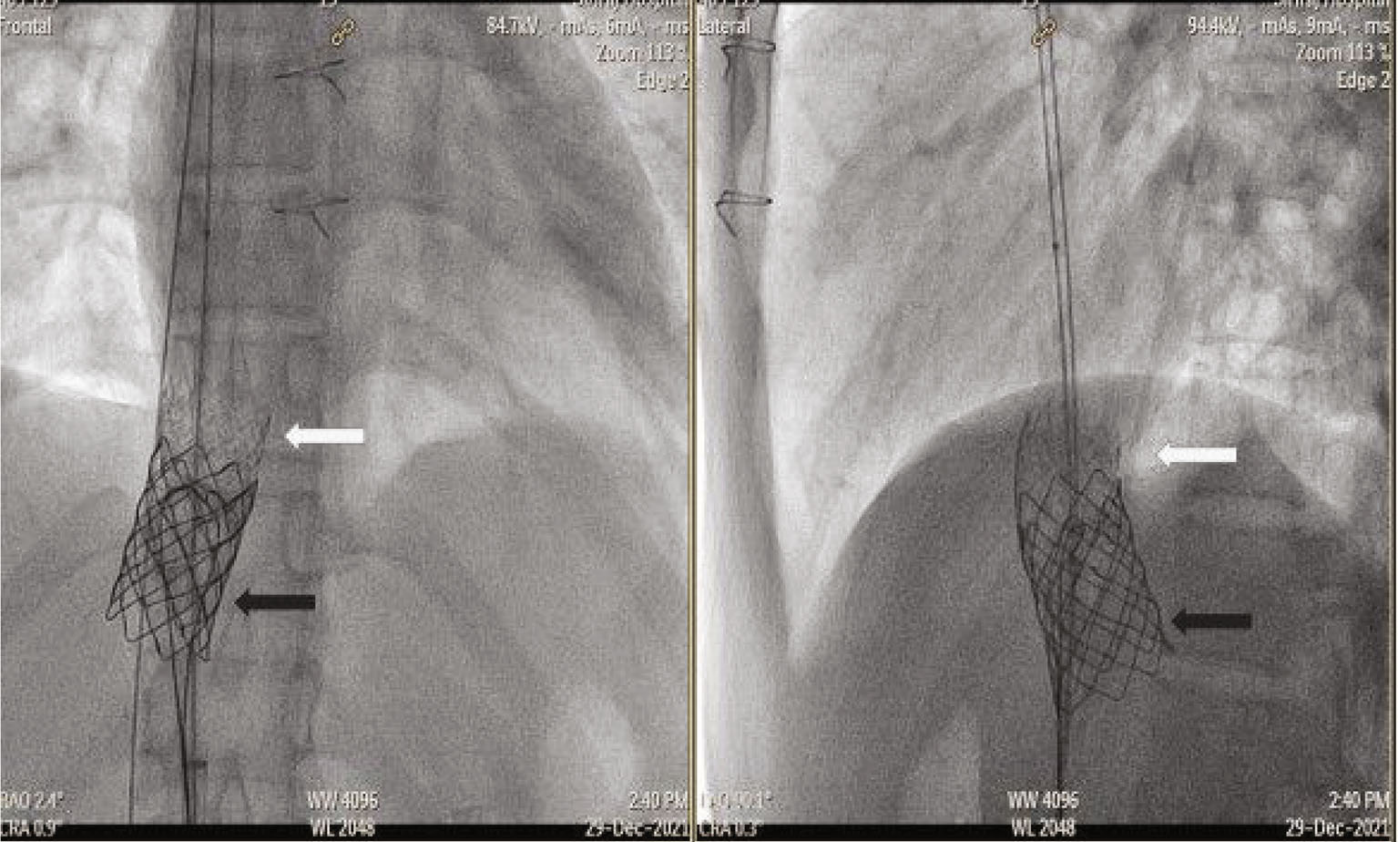
Angiography showing the previously embolized covered stent successfully pulled back inside the primary anchored bare stent. The black arrow and white arrow indicate the position of the bare stent and the covered stent, respectively.

We then deployed a 35 mm Amplatz Gooseneck Snare (Medtronic, Minneapolis, MN, USA) via a delivery catheter in an attempt to grasp distal wire position of from the left femoral vein and was used to snuggle the distal part of the migrated covered stent (Figures 14(a) and 14(b)). The deployed snare was used to squeeze the middle part of the stent with simultaneous inflation of the distal balloon to pull the dislodged stent back into the anchoring bare stent. However, this led to the angulation of the covered stent. We then attempted to snare the stent without balloon inflation, but this strategy was also not successful. We then decided to snare the stent with simultaneous inflation of the distal part of the stent to make it coaxial/telescopic to the anchored bare stent, and this strategy successfully released the migrated stent and allowed us to pull it back into position within the bare stent (Figures 14(c)–14(f)). However, once back inside the bare stent, the snare could not be released from the covered stent. The balloon, covered stent, and snare were then pushed back out of the bare stent. The snare was then repositioned to the distal part of the stent to facilitate successful release of the snare once the covered stent was repositioned within the bare stent (Figures 14(g) and 14(h)). This strategy resulted in the successful repositioning of the covered stent and the successful release of the snare (Figures 15(a) and 15(b)).

**Figure 16:**
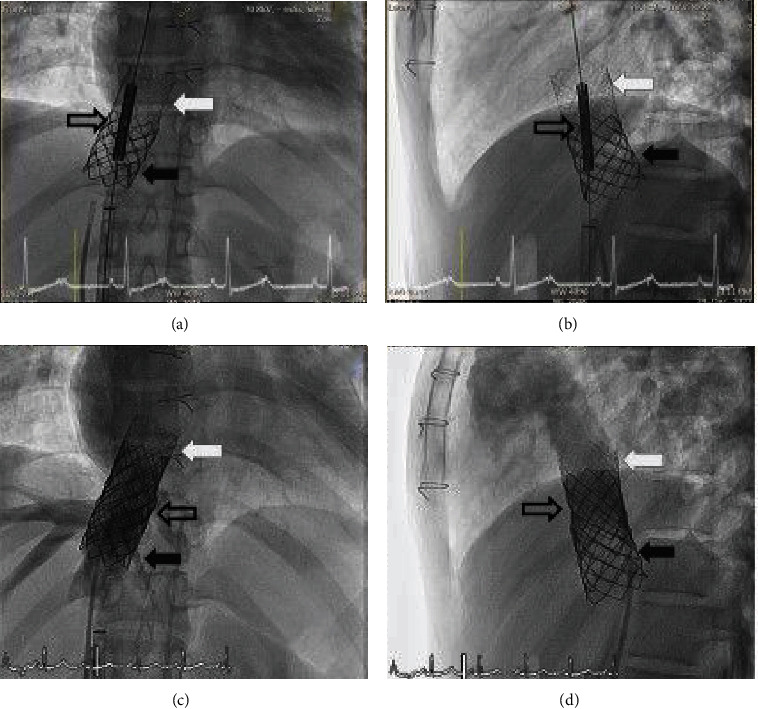
A second bare stent was then applied over the overlapping section of the first bare stent and the covered stent in order to stabilize both the structure of the combination stent and the patency of the affected vasculature ((a) anterior and (b) lateral views on angiography). Postprocedural angiogram showed the second bare stent in place with good blood flow in the hepatic veins ((c) anterior and (d) lateral views on angiography). No contrast was observed filling in the pulmonary vein. The black arrow, white arrow, and black outline arrow indicate the position of the first bare stent, the covered stent, and the second bare stent, respectively.

**Figure 17:**
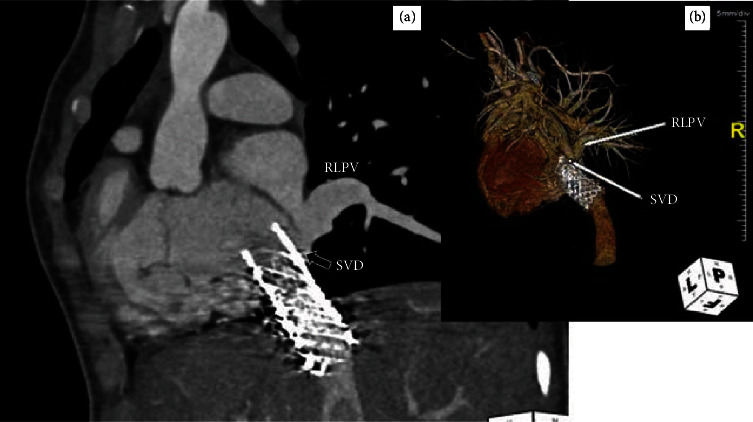
Postprocedural computed tomography angiogram showing (a) a sagittal image and (b) a rendered image of a patent 3-stent structure in the inferior vena cava. The stent structure covers the sinus venosus defect (SVD) with the upper end almost opposed to the interatrial septum causing a small residual opening (black arrow) and unobstructed blood flow from the right lower pulmonary vein (RLPV) to the left atrium (a). The superior segment of the covered stent should be flared by balloon to prevent residual shunt.

A second CP8Z45 bare stent that was mounted on a 22 mm × 5 cm BIB-balloon catheter was then applied over the overlapping section of the first bare stent and the covered stent in order to stabilize both the structure of the combination stent and the patency of the affected vasculature (Figures 16(a) and 16(b)). Postprocedural angiogram showed the second bare stent in place with good blood flow in the hepatic veins and no residual RLPV drainage to the RA (Figures 16(c) and 16(d)). The overall procedure time was 193 minutes, and the fluoroscopy time was 56 minutes. The laboratory results showed normal liver function test. The patient was discharged the following day and aspirin was prescribed. Cardiac CT angiogram (CTA) showed normal RLPV drainage to the LA with a small residual SVD at the superior border of the ASD shunt due to the angulation of the IVC into the interatrial septum (Figure 17). On CTA, both the left and right hepatic veins were patent with no evidence of venous obstruction. A thrombus was suspected in the lower part of the IVC, so oral anticoagulant drug was given. At the 1-month follow-up, duplex ultrasound of the IVC showed complete resolution of the suspected thrombus.

## 3. Discussion

The standard treatment for an SVD with anomalous drainage of pulmonary veins is surgical closure. This surgical procedure yields an excellent complete closure rate; however, some patients develop stenosis of the SVC that requires reoperation. Transcatheter closure of an SVD with PAPVD is a currently available alternative treatment due to its ability to stent the SVC to prevent subsequent stenosis. Several studies reported the success of this procedure using covered stents or device closure in patients having the appropriate anatomy [2–4, 7]. The use of 3-dimensional- (3D-) printed heart models enhances the feasibility of transcatheter closure and promotes improved understanding of the relationship between the structure and surrounding vessels. A 3D-printed heart model can facilitate a simulation of stent or device deployment and can demonstrate successful occlusion of the SVD with redirection of the pulmonary vein to the left atrium [8–10]. The majority of these procedures have been performed in superior SVDs. The first case report of transcatheter closure of an inferior SVD was reported by Kim et al. [6]. Later, He et al. reported a case series of transcatheter closure of inferior SVDs using a device [9]. The challenge of device closure of an inferior SVD is navigating the rim of the atrial septum. In the case series from He et al. [9], all included patients had to have at least two defects, including an inferior SVD. In contrast, the patient profiled in this case report had only one ASD, so device closure was not considered to be an appropriate treatment strategy.

Here, we report the first successful transcatheter closure of an inferior SVD using bare and covered stents. Similar to transcatheter closure of a superior SVD, transcatheter closure of an inferior SVD involves closure of the pulmonary vein defect to redirect pulmonary vein flow to the LA. However, there are four important factors that need to be considered when considering or planning for transcatheter closure of an inferior SVD. First, the position of the hepatic veins needs to be carefully assessed by MRI, CT, or 3D printing before the procedure so that placement of the anchor stent will avoid occlusion of the left and right hepatic veins. Simultaneous venography to ensure that the position of the stent was not too far down during the procedure was also extremely helpful. Second, the angulation of the IVC which pointed more directly to interatrial septum will prevent well alignment of the covered stent than in the SVC showed in the postprocedural CT here that there was a small leakage at the superior rim of the ASD. Third, the effects and severity of intraprocedural complications, such as the stent migration observed in our patient, can be mitigated via intensive preprocedural preparation and a comprehensive inventory of medicines, equipment, and techniques in the cardiac catheterization laboratory. Forth and last, double anti-platelet should be administered to avoid thrombosis of the stents deployed in IVC with slow venous flow.

## 4. Conclusion

Transcatheter closure of an inferior SVD with PAPVD using bare and covered stents in the IVC was shown to be feasible. Careful patient selection and intensive assessment of pulmonary and hepatic vein anatomy before and during the procedure were necessary to achieve a successful outcome. However, a study in a larger patient population with longer-term follow-up is needed to assess the efficacy and safety of this technique.

## References

[1] Abdullah H. A. M., Alsalkhi H. A., Khalid K. A. (2020). Transcatheter closure of sinus venosus atrial septal defect with anomalous pulmonary venous drainage: innovative technique with long-term follow-up. *Catheterization and Cardiovascular Interventions*.

[2] Garg G., Tyagi H., Radha A. S. (2014). Transcatheter closure of sinus venosus atrial septal defect with anomalous drainage of right upper pulmonary vein into superior vena cava--an innovative technique. *Catheterization and Cardiovascular Interventions*.

[3] Riahi M., Velasco Forte M. N., Byrne N. (2018). Early experience of transcatheter correction of superior sinus venosus atrial septal defect with partial anomalous pulmonary venous drainage. *EuroIntervention*.

[4] Rosenthal E., Qureshi S. A., Jones M. (2021). Correction of sinus venosus atrial septal defects with the 10 zig covered Cheatham-platinum stent – an international registry. *Catheterization and Cardiovascular Interventions*.

[5] Abdulwahab H., Husain M. R., Khalid K. A. (2022). Feasibility of transcatheter closure of large secundum atrial septal defect with absent superior or inferior rim. *Journal of Interventional Cardiology*.

[6] Kim H. D., Kim M. S., Yun K. J., Bae S. M., Her S. H., Lee J. H. (2016). Successful transcatheter closure of an inferior sinus venosus atrial septal defect. *The Korean Journal of Internal Medicine*.

[7] Baruteau A. E., Jones M. I., Butera G., Qureshi S. A., Rosenthal E. (2020). Correction percutanee d'une communication interatriale (CIA) sinus venosus avec retour veineux pulmonaire anormal partiel: la procedure de choix chez des patients selectionnes ?. *Archives of Cardiovascular Diseases*.

[8] Thakkar A. N., Chinnadurai P., Breinholt J. P., Lin C. H. (2018). Transcatheter closure of a sinus venosus atrial septal defect using 3D printing and image fusion guidance. *Catheterization and Cardiovascular Diagnosis*.

[9] He L., Cheng G. S., Du Y. J., Zhang Y. S. (2020). Feasibility of device closure for multiple atrial septal defects with an inferior sinus venosus defect: procedural planning using three-dimensional printed models. *Heart, Lung & Circulation*.

[10] Velasco Forte M. N., Byrne N., Valverde I. (2018). Interventional correction of sinus venosus atrial septal defect and partial anomalous pulmonary venous drainage: procedural planning using 3D printed models. *JACC: Cardiovascular Imaging*.

